# The 24-h FEV_1_ time profile of olodaterol once daily via Respimat® and formoterol twice daily via Aerolizer® in patients with GOLD 2–4 COPD: results from two 6-week crossover studies

**DOI:** 10.1186/2193-1801-3-419

**Published:** 2014-08-09

**Authors:** Gregory J Feldman, Jonathan A Bernstein, Alan Hamilton, Michael C Nivens, Lawrence Korducki, Craig LaForce

**Affiliations:** From S. Carolina Pharmaceutical Research, 141 Harold Fleming Court, North Grove Medical Park, Spartanburg, South Carolina 29303 USA; Department of Internal Medicine, Bernstein Clinical Research Center, Cincinnati, Ohio USA; Boehringer Ingelheim, Burlington, Ontario Canada; Boehringer Ingelheim Pharmaceuticals Inc, Biberach an der Riss, Germany; Boehringer Ingelheim Pharma GmbH & Co. KG, Ridgefield, Connecticut USA; North Carolina Clinical Research, Raleigh, North Carolina USA

## Abstract

**Abstract:**

These studies evaluated the 24-h forced expiratory volume in 1 sec (FEV_1_) profile of once-daily (QD) olodaterol compared to placebo and twice-daily (BID) formoterol in patients with moderate to very severe chronic obstructive pulmonary disease. In two replicate, randomized, double-blind, double-dummy, four-way crossover studies, patients received olodaterol 5 and 10 μg QD, formoterol 12 μg BID, or placebo for 6 weeks in addition to usual-care background maintenance therapy. Co-primary end points were FEV_1_ area under the curve from 0–12 h (AUC_0–12_) response (change from baseline) and FEV_1_ AUC from 12–24 h (AUC_12–24_) response after 6 weeks, with FEV_1_ AUC from 0–24 h response identified as a key secondary end point. Other secondary end points included FEV_1_ AUC from 0–3 h and trough FEV_1_ responses, as well as corresponding forced vital capacity responses. With both olodaterol doses, FEV_1_ increased to near-maximal 30 min post-morning dose, which was sustained over 24 h. FEV_1_ also increased within 30 min post-morning dose of formoterol and was sustained over 12 h; the second formoterol dose resulted in a further increase, sustained for an additional 12 h. FEV_1_ AUC_0–12_ and AUC_12–24_ responses with both QD olodaterol doses and BID formoterol were significantly greater than placebo at 6 weeks (*P* < .0001). Secondary end-point outcomes were consistent with those of the co-primary end points. These data, together with those from the wider phase III clinical program, provide evidence for the 24-h bronchodilator efficacy of olodaterol QD in this patient population.

**Trial registry:**

ClinicalTrials.gov; NCT00931385 and NCT00932646.

**Electronic supplementary material:**

The online version of this article (doi:10.1186/2193-1801-3-419) contains supplementary material, which is available to authorized users.

## Introduction

Long-acting bronchodilators, such as long-acting β_2_-agonists (LABAs) and long-acting muscarinic antagonists, are the cornerstone of pharmacologic therapy for patients with chronic obstructive pulmonary disease (COPD) and are considered central to symptom management (Global Initiative for Chronic Obstructive Lung Disease 2013). The first long-acting bronchodilators available for maintenance treatment of COPD were the LABAs salmeterol and formoterol, which had a <24-h duration of action and so required twice-daily (BID) dosing (Global Initiative for Chronic Obstructive Lung Disease 2013). The development of newer LABAs, such as indacaterol, with a longer 24-h duration of action (Rodrigo & Neffen 2012) allows for a once-daily (QD) posology (Toy et al. 2011).

Olodaterol is a LABA (Bouyssou et al. 2010a) with high β_2_-receptor selectivity and a near full agonist response at the human β_2_-adrenoceptor (Bouyssou et al. 2010b). Effective 24-h bronchodilation with olodaterol in both asthma and COPD has been confirmed by single-dose studies (van Noord et al. 2011; O’Byrne et al. 2009) and studies over 4 weeks (Joos et al. 2012; O’Byrne et al. 2012; van Noord et al. 2009). The results of these phase II studies provided the rationale to further investigate 5 and 10 μg QD doses of olodaterol in a phase III clinical program.

The comprehensive olodaterol phase III clinical program was designed to evaluate multiple efficacy and safety end points in five sets of paired studies that between them assessed 48-week lung-function efficacy, symptomatic benefit, 24-h bronchodilator profile, and exercise capacity. All studies were conducted in replicate to independently authenticate outcomes (US Department of Health and Human Services et al. 1998).

The primary objective of the replicate studies presented here was to determine the 24-h forced expiratory volume in 1 sec (FEV_1_) profile of olodaterol 5 and 10 μg QD in comparison to placebo and formoterol 12 μg BID in patients with moderate to very severe (Global initiative for chronic Obstructive Lung Disease stage 2–4) COPD. Formoterol was chosen as the active comparator because QD LABAs were not available at the time these studies were conducted. These studies are complementary to two replicate pivotal studies that, as a secondary end point, measured FEV_1_ responses over 12 h in a subset of patients (NCT00782210 and NCT00782509) (Ferguson et al. 2013) and two replicate 6-week studies (NCT01040689 and NCT01040728) that assessed the 24-h profile of olodaterol QD vs tiotropium (Lange et al. 2013).

In the studies presented here, the study population was chosen to allow evaluation of the 24-h bronchodilation activity of olodaterol in patients closely representative of those in clinical practice, with specific attention given to disease severity, co-morbidities, and background therapies (European Medicines Agency 2012).

## Methods

### Study design

**Figure 1:**
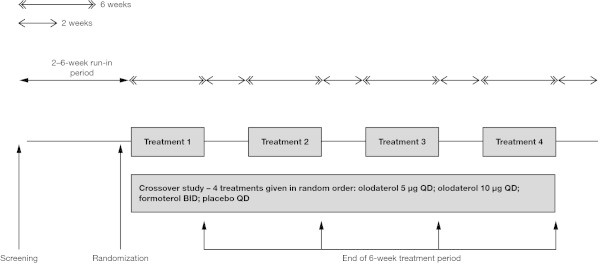
**Study design.** BID = twice daily; QD = once daily.

These were replicate, multicenter, randomized, double-blind, double-dummy, placebo-controlled, four-way crossover studies (registered with ClinicalTrials.gov: NCT00931385 [study 1222.24] and NCT00932646 [study 1222.25]) conducted in the US (Figure 1). Eligible patients who successfully completed a 2- to 6-week run-in period to ensure clinical stability received each of the following treatments in a random sequence: olodaterol 5 μg QD, olodaterol 10 μg QD, formoterol 12 μg BID, and placebo. Each administration of olodaterol comprised two actuations of the Respimat® inhaler QD, while formoterol was administered via Aerolizer® with each administration comprising one actuation BID. Each treatment period lasted for 6 weeks, with a 14-day washout in between. Patients were evaluated for 14 days following study completion. With the exception of LABAs, patients continued usual-care background COPD maintenance treatment, including short-acting muscarinic antagonists, long-acting muscarinic antagonists, inhaled corticosteroids, and xanthines, throughout the duration of these trials. Patients on LABAs were allowed to switch to short-acting muscarinic antagonists. Salbutamol (100 μg) was provided to all patients as rescue medication.

The study was approved by local ethics committees and carried out according to the Declaration of Helsinki and local regulations. Prior to study initiation, the protocol was approved by the local Institutional Review Board, Independent Ethics Committee, and the Competent Authority. All patients provided written, informed consent prior to the study commencing. Details of the local Institutional Review Boards are provided in Additional file 1: Table S1.

### Patients

Patients were enrolled into the study if they met the following inclusion criteria: aged ≥40 years; current or ex-smokers with a smoking history of >10 pack-years; post-bronchodilator FEV_1_ < 80% of predicted normal; and post-bronchodilator FEV_1_/forced vital capacity (FVC) <70%. Key exclusion criteria were: significant disease other than COPD (defined by the investigator as a disease that may put the patient at risk by participating in the study, influence study outcomes, or cause concern with regards to the patient’s ability to participate in the study); history of asthma; history of myocardial infarction within 1 year of the screening visit; and unstable or life-threatening cardiac arrhythmia within the past year.

### Study outcomes

The primary objective of the study was to determine if olodaterol 5 and 10 μg QD administered via the Respimat® inhaler provided superior 24-h bronchodilation vs placebo. A secondary objective was to compare the 24-h FEV_1_ time profile of QD olodaterol with that of BID formoterol. Co-primary end points were FEV_1_ area under the curve from 0 to 12 h (AUC_0–12_) response (defined as change from study baseline) and FEV_1_ AUC from 12 to 24 h (AUC_12–24_) response after 6 weeks of treatment. FEV_1_ AUC from 0 to 24 h (AUC_0–24_) response was identified as a key secondary end point. Other secondary efficacy variables included FEV_1_ measurements at individual time points over 24 h after 6 weeks of treatment, FEV_1_ AUC from 0 to 3 h (AUC_0–3_) response, peak FEV_1_ response, and trough FEV_1_ response. Corresponding FVC responses after 6 weeks were also measured. Safety end points included adverse events (AEs), vital signs, blood chemistry, and electrocardiogram.

### Assessments

All qualifying pulmonary function tests (PFTs) (FEV_1_ and FVC) were conducted during the screening visit, and were started at approximately the same time of day for each patient (ie, between 7:00 AM and 9:00 AM; ±30 min maximal difference between the start of the tests on visit 2 and those conducted on subsequent test days). At the start of each treatment period, PFTs were conducted 60 min and 10 min before administration of the morning dose of study drug and at 30 min, 1, 2, and 3 h post-morning dose. Further PFTs were carried out at the end of each treatment period 30 min before administration of the morning dose and at 30 min, 1, 2, 3, 4, 6, 8, 10 h, 11 h 50 min, 12 h 30 min, and 13, 14, 22, 23 h, and 23 h 50 min post-morning dose of study drug (the evening dose of study drug was administered 12 h after the morning dose). Patients were required to stay overnight in the clinic or at a nearby hotel to ensure the quality and timing of PFTs at 22, 23 h, and 23 h 50 min post-dose on the second day of the 24-h PFT visit. All spirometric maneuvers were conducted in triplicate and performed according to American Thoracic Society/European Respiratory Society criteria (Miller et al. 2005). Daily trial medication and rescue medication use were recorded in paper diaries.

Safety end points were assessed in all patients who received at least one dose of study drug. All AEs, irrespective of causality, were monitored and recorded at each visit.

### Statistical analysis

A sample size of 80 randomized patients provided 90% power to detect a treatment difference between olodaterol and placebo of 60 mL in FEV_1_ AUC_0–12_ and 51 mL in FEV_1_ AUC_12–24_, based on an estimated standard deviation of 0.160 and 0.140 L, respectively. The conservative randomized discontinuation was estimated to be 20%, resulting in 100 patients randomized.

The primary and secondary efficacy end points were based on the full analysis set, which included all patients with baseline data and evaluable post-dosing data for at least the first co-primary end point. Both primary and secondary end points were analyzed using a mixed-effects repeated measures model with terms for “center”, “patient within center”, “treatment”, and “period”. Analyses included the fixed categorical effects of “treatment”, “period”, and “random effect for patient”. Compound symmetry covariance structure was used to model within-patient variation. Analyses of AEs, laboratory data, and vital signs were descriptive in nature.

## Results

### Patient disposition and baseline characteristics

**Figure 2:**
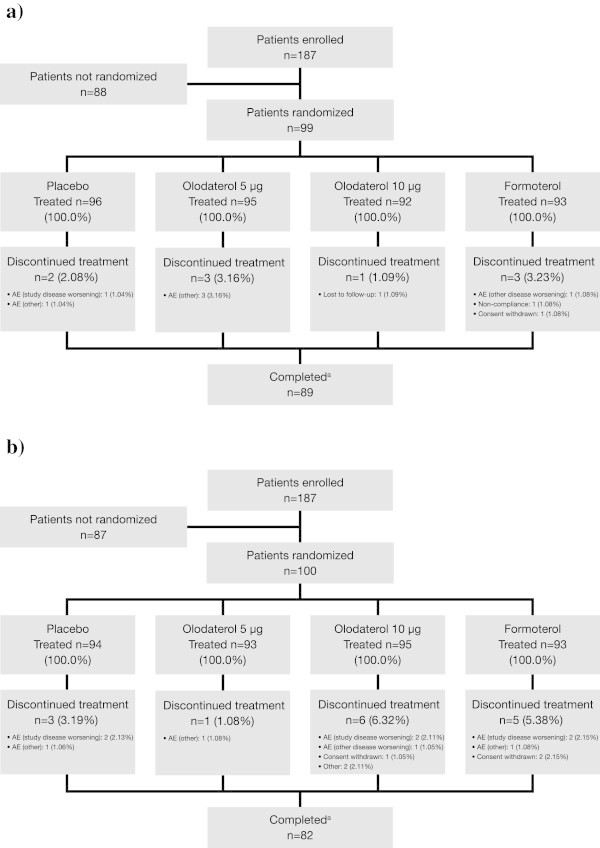
**CONSORT diagram illustrating participant flow in (a) study 1222.24 and (b) study 1222.25.** AE = adverse event. ^a^All patients who completed 4 treatment periods.

A total of 199 patients were randomized to treatment in both studies (Figure 2): 99 in study 1222.24 and 100 in study 1222.25. All patients were randomized between August 31 and September 15, 2009 (1222.24), and September 01 to 15, 2009 (1222.25) at different US sites involved in each study. All randomized patients received at least one dose of study drug and the majority (86%) completed all four treatment periods. There was a total of 24 occurrences of a patient discontinuing a treatment period (nine in study 1222.24 and 15 in study 1222.25), primarily due to AEs. Patients who discontinued from a treatment period were permitted to remain in the study and continue into the next treatment period. Patient demographics and baseline disease characteristics were well balanced across the studies (Table 1).

**Table 1:** **Baseline patient demographics and disease characteristics (treated set)**

	Study 1222.24	Study 1222.25
	(n = 99)	(n = 100)
Sex, n (%)		
Male	52 (52.5)	54 (54.0)
Female	47 (47.5)	46 (46.0)
Age, mean (SD), years	61.8 (8.9)	63.5 (8.2)
COPD diagnosis, mean (SD), years	7.4 (5.2)	9.4 (7.9)
Pre-bronchodilator		
Mean (SD) FEV_1_, L	1.241 (0.451)	1.242 (0.504)
Mean (SD) FEV_1_/FVC, %	49.571 (11.562)	48.673 (12.144)
Mean (SD) % of predicted normal FEV_1_	44.904 (13.908)	46.010 (14.678)
Post-bronchodilator		
Mean (SD) FEV_1_, L	1.417 (0.494)	1.439 (0.530)
Mean (SD) FEV_1_ change from		
pre-bronchodilator, L	0.177 (0.158)	0.197 (0.158)
Mean (SD) FEV_1_/FVC, %	50.224 (11.133)	49.354 (11.460)
Mean (SD) % of predicted normal FEV_1_	51.368 (15.009)	53.242 (14.706)
GOLD stage, n (%)		
2	51 (51.5)	56 (56.0)
3	39 (39.4)	39 (39.0)
4	9 (9.1)	5 (5.0)
BMI, mean (SD), kg/m^2^	28.7 (7.7)	27.8 (7.2)
Current smoker, n (%)	60 (60.6)	43 (43.0)
Smoking history, mean (SD), pack-years	54.9 (24.8)	51.2 (26.7)

BMI = body mass index; COPD = chronic obstructive pulmonary disease; FEV_1_ = forced expiratory volume in 1 sec; FVC = forced vital capacity; GOLD = Global initiative for chronic Obstructive Lung Disease; SD = standard deviation.

### Efficacy

**Figure 3:**
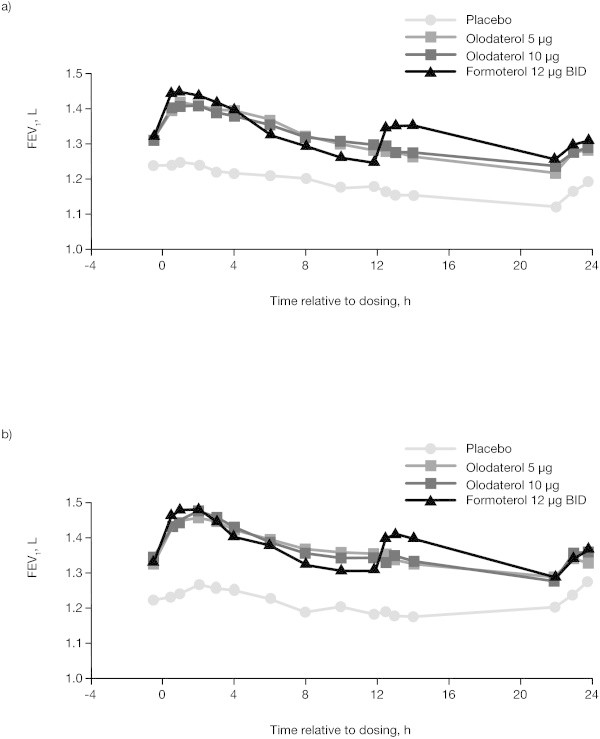
**FEV**_**1**_ **24-h profiles of olodaterol 5 and 10 μg and formoterol 12 μg BID compared to placebo at week 6 in (a) study 1222.24 and (b) study 1222.25.** BID = twice daily; FEV_1_ = forced expiratory volume in 1 sec.

The FEV_1_ time profiles for both doses of olodaterol were similar over 24 h (Figure 3). Mean FEV_1_ increased to near-maximal within 30 min and was sustained over the full 24-h post-dose evaluation period. Following the morning dose of formoterol, mean FEV_1_ also increased within 30 min and was comparable to both doses of olodaterol 0 to 3 h post-dose. The FEV_1_ time profile of formoterol intersected with the FEV_1_ time profile of both olodaterol doses at 4 h and was lower than the FEV_1_ responses observed with both doses of olodaterol 4 to 12 h post-dose. The evening dose of formoterol resulted in an additional increase in adjusted mean FEV_1_, which was sustained over the 12 to 24-h period (Figure 3).

### Primary and key secondary end points

In both studies, both primary end points of FEV_1_ AUC_0–12_ and AUC_12–24_ responses and the key secondary end point of FEV_1_ AUC_0–24_ response were significantly improved with olodaterol 5 μg QD, olodaterol 10 μg QD, and formoterol 12 μg BID compared to placebo (*P* < .0001) (Table 2). Pooled data showed no differences between olodaterol 5 and 10 μg QD compared to formoterol 12 μg BID for the FEV_1_ AUC_0–12_ response. However, the adjusted mean FEV_1_ AUC_12–24_ response for formoterol 12 μg BID was significantly greater than olodaterol 5 and 10 μg QD. For both FEV_1_ AUC_0–12_ and AUC_12–24_ responses, both doses of olodaterol were similar (Table 3). No statistically significant differences in FEV_1_ AUC_0–24_ responses were reported between all three active comparators (Table 3).

**Table 2:** **Adjusted mean FEV**_**1**_ **AUC**_**0–12**_ **, AUC**_**12–24**_ **, and AUC**_**0–24**_ **responses (L) compared to placebo after 6 weeks**

	Treatment		Adjusted ^a^mean (95% CI) difference from placebo at 6 weeks		
***FEV***_***1***_ ***AUC***_***0–12***_	n	Adjusted mean (SE)	Mean (SE)	***P***value	95% CI
Study 1222.24					
Placebo	93	−0.060 (0.020)			
Olodaterol 5 μg QD	92	0.088 (0.021)	0.148 (0.018)	< .0001	0.113, 0.183
Olodaterol 10 μg QD	91	0.088 (0.021)	0.148 (0.018)	< .0001	0.113, 0.183
Formoterol 12 μg BID	90	0.081 (0.021)	0.141 (0.018)	< .0001	0.106, 0.177
Study 1222.25					
Placebo	91	−0.022 (0.024)			
Olodaterol 5 μg QD	92	0.150 (0.024)	0.172 (0.017)	< .0001	0.139, 0.205
Olodaterol 10 μg QD	90	0.152 (0.024)	0.174 (0.017)	< .0001	0.140, 0.208
Formoterol 12 μg BID	90	0.136 (0.024)	0.158 (0.017)	< .0001	0.124, 0.191
*FEV*_*1*_ *AUC*_*12–24*_					
Study 1222.24					
Placebo	93	−0.123 (0.021)			
Olodaterol 5 μg QD	92	−0.014 (0.022)	0.109 (0.019)	< .0001	0.073, 0.146
Olodaterol 10 μg QD	91	0.004 (0.022)	0.127 (0.019)	< .0001	0.091, 0.164
Formoterol 12 μg BID	90	0.049 (0.022)	0.172 (0.019)	< .0001	0.135, 0.209
Study 1222.25					
Placebo	91	−0.048 (0.025)			
Olodaterol 5 μg QD	92	0.069 (0.025)	0.118 (0.018)	< .0001	0.082, 0.154
Olodaterol 10 μg QD	90	0.072 (0.025)	0.120 (0.018)	< .0001	0.084, 0.157
Formoterol 12 μg BID	90	0.107 (0.025)	0.155 (0.018)	< .0001	0.119, 0.191
*FEV*_*1*_ *AUC*_*0–24*_					
Study 1222.24					
Placebo	93	−0.092 (0.020)			
Olodaterol 5 μg QD	92	0.037 (0.021)	0.128 (0.017)	< .0001	0.094, 0.163
Olodaterol 10 μg QD	91	0.046 (0.021)	0.137 (0.017)	< .0001	0.103, 0.172
Formoterol 12 μg BID	90	0.065 (0.021)	0.156 (0.018)	< .0001	0.122, 0.191
Study 1222.25					
Placebo	91	−0.035 (0.024)			
Olodaterol 5 μg QD	92	0.110 (0.024)	0.145 (0.016)	< .0001	0.114, 0.176
Olodaterol 10 μg QD	90	0.112 (0.024)	0.147 (0.016)	< .0001	0.116, 0.179
Formoterol 12 μg BID	90	0.121 (0.024)	0.156 (0.016)	< .0001	0.125, 0.187

AUC_0–12_ = area under the curve from 0 to 12 h; AUC_0–24_ = area under the curve from 0 to 24 h; AUC_12–24_ = area under the curve from 12 to 24 h; BID = twice daily; FEV_1_ = forced expiratory volume in 1 sec; SE = standard error.

^a^Based on a mixed effects repeated measures model. The model includes treatment and period as fixed effects and center and patient within center as random effects, along with compound symmetry as a covariance structure for within−patient variation.

**Table 3:** **Adjusted mean FEV**_**1**_ **AUC**_**0–12**_ **, FEV**_**1**_ **AUC**_**12–24**_ **, and FEV**_**1**_ **AUC**_**0–24**_ **responses (L); comparisons across active treatment arms after 6 weeks (pooled analysis)**

	Treatment difference					
	FEV _1_AUC _0–12_	***P***value	FEV _1_AUC _12–24_	***P***value	FEV _1_AUC _0–24_	***P***value
	Mean (SE)		Mean (SE)		Mean (SE)	
Olodaterol 10 μg QD vs 5 μg QD	0.001 (0.012)	.9527	0.010 (0.013)	.4423	0.005 (0.012)	.6474
Olodaterol 10 μg QD vs formoterol 12 μg BID	0.011 (0.012)	.3588	−0.040 (0.013)	.0024	−0.014 (0.012)	.2268
Olodaterol 5 μg QD vs formoterol 12 μg BID	0.011 (0.012)	.3876	−0.050 (0.013)	.0001	−0.020 (0.012)	.0944

AUC_0–12_ = area under the curve from 0 to 12 h; AUC_0–24_ = area under the curve from 0 to 24 h; AUC_12–24_ = area under the curve from 12 to 24 h; BID = twice daily; FEV_1_ = forced expiratory volume in 1 sec; QD = once daily; SE = standard error.

### Secondary end points

There were statistically significant improvements in the peak FEV_1_ response for all active comparators compared to placebo (*P* < .0001) (Table 4). Pooled analysis demonstrated that there were no statistically significant differences in peak FEV_1_ responses between the two olodaterol doses; however, peak FEV_1_ response for both olodaterol 5 and 10 μg doses was significantly lower than formoterol (−0.036 and −0.034 L, respectively). Additionally, significant improvements in trough FEV_1_ and FEV_1_ AUC_0–3_ responses were observed with both doses of olodaterol and formoterol in comparison to placebo (Table 4).

**Table 4:** **Adjusted mean FEV**_**1**_ **AUC**_**0–3**_ **, peak FEV**_**1**_ **, and trough FEV**_**1**_ **responses (L) compared to placebo after 6 weeks**

	Treatment		Adjusted ^a^mean (95% CI) difference from placebo at 6 weeks		
***FEV***_***1***_ ***AUC***_***0–3***_	n	Adjusted mean (SE)	Mean (SE)	***P***value	95% CI
Study 1222.24					
Placebo	93	−0.030 (0.020)			
Olodaterol 5 μg QD	92	0.134 (0.021)	0.164 (0.019)	< .0001	0.126, 0.201
Olodaterol 10 μg QD	91	0.135 (0.021)	0.164 (0.019)	< .0001	0.127, 0.202
Formoterol 12 μg BID	90	0.168 (0.021)	0.198 (0.019)	< .0001	0.160, 0.236
Study 1222.25					
Placebo	91	0.004 (0.024)			
Olodaterol 5 μg QD	92	0.190 (0.025)	0.186 (0.019)	< .0001	0.149, 0.223
Olodaterol 10 μg QD	90	0.202 (0.025)	0.198 (0.019)	< .0001	0.162, 0.235
Formoterol 12 μg BID	90	0.217 (0.025)	0.213 (0.019)	< .0001	0.176, 0.250
*Peak FEV*_*1*_					
Study 1222.24					
Placebo	93	0.034 (0.022)			
Olodaterol 5 μg QD	92	0.208 (0.022)	0.174 (0.020)	< .0001	0.135, 0.214
Olodaterol 10 μg QD	91	0.200 (0.022)	0.166 (0.020)	< .0001	0.127, 0.206
Formoterol 12 μg BID	90	0.251 (0.022)	0.218 (0.020)	< .0001	0.178, 0.257
Study 1222.25					
Placebo	91	0.076 (0.026)			
Olodaterol 5 μg QD	92	0.268 (0.026)	0.192 (0.019)	< .0001	0.154, 0.230
Olodaterol 10 μg QD	90	0.273 (0.026)	0.197 (0.020)	< .0001	0.158, 0.235
Formoterol 12 μg BID	90	0.293 (0.026)	0.217 (0.020)	< .0001	0.178, 0.255
*Trough FEV*_*1*_					
Study 1222.24					
Placebo	93	−0.093 (0.023)			
Olodaterol 5 μg QD	92	0.012 (0.024)	0.106 (0.021)	< .0001	0.064, 0.147
Olodaterol 10 μg QD	91	0.020 (0.024)	0.113 (0.021)	< .0001	0.072, 0.155
Formoterol 12 μg BID	90	0.040 (0.024)	0.133 (0.021)	< .0001	0.092, 0.175
Study 1222.25					
Placebo	91	0.012 (0.030)			
Olodaterol 5 μg QD	92	0.109 (0.030)	0.097 (0.026)	.0003	0.045, 0.148
Olodaterol 10 μg QD	90	0.115 (0.030)	0.103 (0.026)	.0001	0.051, 0.155
Formoterol 12 μg BID	90	0.093 (0.030)	0.080 (0.026)	.0026	0.028, 0.132

AUC_0–3_ = area under the curve from 0 to 3 h; BID = twice daily; FEV_1_ = forced expiratory volume in 1 sec; QD = once daily; SE = standard error.

^a^Based on a mixed effects repeated measures model. The model includes treatment and period as fixed effects and center and patient within center as random effects, along with compound symmetry as a covariance structure for within−patient variation.

The corresponding FVC responses after 6 weeks’ treatment with olodaterol 5 and 10 μg QD and formoterol 12 μg BID were consistent with the FEV_1_ AUC responses and significantly improved vs placebo (Additional file 1: Tables S2–S4). In the pooled analysis, no statistically significant differences between olodaterol doses were observed for FVC AUC_0–12_, FVC AUC_12–24_, and FVC AUC_0–24_. Similar to FEV_1_ AUC_12–24_, the adjusted mean FVC AUC_12–24_ response for formoterol 12 μg BID was significantly greater than olodaterol 5 and 10 μg QD (−0.083 L [*P* = .0001] and −0.074 L [*P* = .0008], respectively, vs formoterol 12 μg BID). There were no other statistically significant differences in FVC AUC responses between the active treatment groups. FVC AUC_0–12_, AUC_12–24_, AUC_0–24_, and peak and trough FVC responses are shown in Additional file 1: Tables S2–S6, respectively.

### Safety

Overall, 129 patients (64.8%) reported at least one AE during the studies. Incidence of AEs across active treatment groups was comparable. A total of 13 patients had AEs that were considered by the investigator to be related to study drug. The most frequently reported treatment-emergent AEs were COPD (17.6%) and upper respiratory tract infection (9.5%) (Table 5). Investigator-defined related AEs for each treatment group are shown in Additional file 1: Table S7. In total, 23 patients across both studies reported at least one serious AE, with the most frequently reported being COPD (three patients, study 1222.24; four patients, study 1222.25). Serious AEs in each treatment group are shown in Additional file 1: Table S7. One death in each study was reported: cardio-respiratory arrest (olodaterol 5 μg, study 1222.24) and respiratory failure (olodaterol 10 μg, study 1222.25). These were not considered by the investigator to be related to study treatment.

**Table 5:** **Frequency of AEs (pooled analysis)**

	Pooled analysis				
	Placebo	Olodaterol 5 μg	Olodaterol 10 μg	Formoterol 18 μg	Total
	(n = 190)	(n = 188)	(n = 187)	(n = 186)	(n = 199)
Patients with any AE	60 (31.6)	61 (32.4)	64 (34.2)	49 (26.3)	129 (64.8)
Patients with severe AEs	11 (5.8)	9 (4.8)	6 (3.2)	10 (5.4)	28 (14.1)
Discontinuations due to AEs	4 (2.1)	4 (2.1)	3 (1.6)	3 (1.6)	14 (7.0)
COPD	12 (6.3)	10 (5.3)	9 (4.8)	8 (4.3)	35 (17.6)
Upper respiratory tract infection	4 (2.1)	5 (2.7)	7 (3.7)	7 (3.8)	19 (9.5)
Bronchitis	3 (1.6)	7 (3.7)	4 (2.1)	4 (2.2)	14 (7.0)
Cough	1 (0.5)	6 (3.2)	1 (0.5)	4 (2.2)	12 (6.0)
Headache	2 (1.1)	2 (1.1)	5 (2.7)	2 (1.1)	9 (4.5)
Sinusitis	3 (1.6)	1 (0.5)	3 (1.6)	3 (1.6)	9 (4.5)
Urinary tract infection	4 (2.1)	0	4 (2.1)	2 (1.1)	9 (4.5)
Diarrhea	1 (0.5)	0	2 (1.1)	2 (1.1)	5 (2.5)
Nausea	3 (1.6)	1 (0.5)	0	1 (0.5)	5 (2.5)
Muscle spasms	1 (0.5)	1 (0.5)	1 (0.5)	2 (1.1)	5 (2.5)
Chest pain	2 (1.1)	1 (0.5)	2 (1.1)	0	5 (2.5)
Pneumonia	1 (0.5)	0	2 (1.1)	1 (0.5)	4 (2.0)
Respiratory tract congestion	3 (1.6)	0	1 (0.5)	0	4 (2.0)

AE = adverse event; COPD = chronic obstructive pulmonary disease.

No changes indicative of an AE were observed for any laboratory parameters or vital signs with either dose of olodaterol, or formoterol.

## Discussion

These replicate studies were designed to complement the evidence of long-term efficacy and safety provided by the pivotal 48-week studies in the olodaterol clinical trial program by evaluating the full 24-h FEV_1_ time profile of olodaterol 5 and 10 μg QD in comparison to placebo and formoterol 12 μg BID after chronic dosing.

FEV_1_ AUC_0–12_ and AUC_12–24_ were chosen as co-primary end points to allow a comparison between the different dosing regimens of olodaterol QD and formoterol BID. FEV_1_ AUC_0–24_ was included as a key secondary end point as it offered a comparison of the average 24-h FEV_1_ response between the active comparators.

This evaluation demonstrated that FEV_1_ AUC_0–12_, AUC_12–24_, and AUC_0–24_ responses were all significantly improved with both doses of olodaterol QD and formoterol BID vs placebo. There were distinct differences in the profiles of olodaterol and formoterol over the 24-h dosing interval, as might be expected given the different durations of action and consequent variations in dosing frequency; it should be noted that the methodology used likely overestimates the differences between active treatments. Nevertheless, FEV_1_ AUC_0–24_, a reflection of the mean bronchodilator effect over 24 h, was similar for both doses of olodaterol and formoterol. Similar differences in the 24-h FEV_1_ time profiles between QD and BID muscarinic antagonists have recently been observed in a trial comparing the 24-h bronchodilatory efficacy of aclidinium BID vs tiotropium QD in patients with moderate to severe COPD (Beier et al. 2013).

While these replicate studies measured lung function over a continuous 24-h dosing interval, there was a necessary pause in testing between 14 and 22 h post-dose to allow patients to have a relatively full night’s sleep. As such, it is to be noted that the calculation of FEV_1_ AUC_12–24_ in the study assumes a linear slope between 14 and 22 h post-dose for both olodaterol and formoterol. The FEV_1_ time profiles in Figure 3 clearly show a separation of formoterol and olodaterol as a result of the second peak for formoterol, 1 to 2 h after the evening dose. In contrast, between 22 and 24 h post-dose, the FEV_1_ time profiles for olodaterol and formoterol have converged. Due to the necessary pause in lung-function testing between 14 and 22 h post-dose, to allow patients to sleep, the precise time point at which this convergence occurred is not known.

The results for all other secondary outcomes supported those of the primary end points, with FVC responses mirroring FEV_1_ outcomes. The inclusion of peak FEV_1_ AUC_0–3_ and trough FEV_1_ measurements (at both ends of the daily dosing profile) provided further evidence to confirm the 24-h activity of olodaterol, with the ratio reflecting the degree of bronchodilation that is maintained at the end of the dosing interval in relation to the peak bronchodilation observed in the first hours after dosing. In addition, all FEV_1_ and FVC responses observed were in line with those expected for a patient population continuing with standard bronchodilation and corticosteroid maintenance therapy.

The outcomes from these replicate studies support those from earlier phase II trials employing similar end points, which demonstrated that single doses of olodaterol 5 and 10 μg QD provided effective and significant bronchodilation over a 24-h period (Joos et al. 2012; van Noord et al. 2009). Furthermore, the outcomes from these replicate studies add to the comprehensive set of evidence for the efficacy and safety of olodaterol QD in patients with COPD derived from the wider olodaterol phase III clinical program. A similar 12-h bronchodilation profile for olodaterol was observed in a subset of patients from two independent, 48-week, pivotal studies of olodaterol 5 and 10 μg QD in comparison with placebo (Ferguson et al. 2013). Additionally, outcomes from similar phase III, replicate, 6-week studies demonstrated that olodaterol 5 and 10 μg QD significantly improved FEV_1_ AUC_0–12_ and AUC_12–24_ responses compared to placebo, with a 24-h bronchodilator profile comparable to tiotropium (Lange et al. 2013).

A question that arises from the difference in the 24-h lung-function profiles of olodaterol QD and formoterol BID is whether the second evening peak in FEV_1_ with formoterol is associated with any improvements in night-time symptoms compared to olodaterol. This cannot be determined from our studies as no assessment of daytime and night-time symptoms was performed. However, two long-term, 48-week studies within the olodaterol phase III program were conducted using the BID comparator formoterol (Koch et al. 2013). Despite the second dose of formoterol being given in the evening, there were no differences between active treatments in night-time rescue medication usage at any time point in these studies (Boehringer Ingelheim, data on file). These data suggest that the evening peak in lung function with formoterol was not manifested in terms of advantages in night-time symptomatology.

Treatment with both doses of olodaterol and formoterol was well tolerated and incidence of AEs across treatment groups was comparable with the most commonly reported AEs: COPD and upper respiratory tract infection. In addition, safety outcomes in these studies were consistent with those reported in the pivotal studies.

## Conclusions

These data, together with those from the wider phase III clinical program, provide evidence for the 24-h bronchodilator efficacy of olodaterol QD in patients with moderate to very severe COPD, with no differences in efficacy and tolerability observed between olodaterol 5 and 10 μg QD. Results from this study support the selection of the 5 μg dose for later use in clinical practice.

## Electronic supplementary material

Additional file 1: **Supplementary tables S1 to S7.** (PDF 40 KB)

## Abbreviations

AE: Adverse event
AUC_0–3_: Area under the curve from 0 to 3 h
AUC_0–12_: Area under the curve from 0 to 12 h
AUC_0–24_: Area under the curve from 0 to 24 h
AUC_12–24_: Area under the curve from 12 to 24 h
BID: Twice daily
COPD: Chronic obstructive pulmonary disease
FEV_1_: Forced expiratory volume in 1 sec
FVC: Forced vital capacity
LABA: Long-acting β_2_-agonist
QD: Once daily
PFT: Pulmonary function test.
