# Supplementary material for: The 24-h FEV1 time profile of olodaterol once daily via Respimat® and formoterol twice daily via Aerolizer® in patients with GOLD 2–4 COPD: results from two 6-week crossover studies
Source: Springerplus. 2014 Aug 9;3:419. doi: 10.1186/2193-1801-3-419 (PMC4152473; doi:10.1186/2193-1801-3-419)
Supplement: Supplementary file 1 — Additional file 1: Supplementary tables S1 to S7. (PDF 40 KB) [file 40064_2014_1159_MOESM1_ESM.pdf]

Additional file 1

Table S1 IRBs

Study 1222.24

IRB (name/address)	IRB Chairperson	Centre number(s)/ Investigator(s)	Protocol/Amendments	Date of final approval
Schulman Associates, IRB, 4290 Glendale–Milford Rd, Cincinnati, OH 45242	Sharon Nelson, MSN, RN, CNS	24002 Jonathan A Bernstein, MD	1222.24 and Amendment 01	05/May/2009 25/Aug/2009
Schulman Associates, IRB, 4290 Glendale–Milford Rd, Cincinnati, OH 45242	Sharon Nelson, MSN, RN, CNS	24003 Joseph Boscia III, MD, CPI	1222.24 and Amendment 01	29/Apr/2009 25/Aug/2009

Schulman Associates, IRB, 4290 Glendale–Milford Rd, Cincinnati, OH 45242	Sharon Nelson, MSN, RN, CNS	24006 David R. Erb, MD	1222.24 and Amendment 01	29/Apr/2009 25/Aug/2009
Schulman Associates, IRB, 4290 Glendale–Milford Rd, Cincinnati, OH 45242	Sharon Nelson, MSN, RN, CNS	24001 Gregory J. Feldman, MD, CPI	1222.24 and Amendment 01	04/May/2009 25/Aug/2009
Schulman Associates, IRB, 4290 Glendale–Milford Rd, Cincinnati, OH 45242	Sharon Nelson, MSN, RN, CNS	24009 Steven G. Hull, MD	1222.24 and Amendment 01	29/Apr/2009 25/Aug/2009
Schulman Associates, IRB, 4290 Glendale–Milford Rd, Cincinnati, OH 45242	Sharon Nelson, MSN, RN, CNS	24010 Norman K. Imes, MD	1222.24 and Amendment 01	04/Jun/2009 25/Aug/2009

Schulman Associates, IRB, 4290 Glendale–Milford Rd, Cincinnati, OH 45242	Sharon Nelson, MSN, RN, CNS	24004<br>Andras Koser, MD, MBA, CCPI	1222.24 and<br>Amendment 01	30/Apr/2009<br>25/Aug/2009
Schulman Associates, IRB, 4290 Glendale–Milford Rd, Cincinnati, OH 45242	Sharon Nelson, MSN, RN, CNS	24011<br>Lon D. Lynn, DO	1222.24 and<br>Amendment 01	30/Apr/2009<br>25/Aug/2009
Schulman Associates, IRB, 4290 Glendale–Milford Rd, Cincinnati, OH 45242	Sharon Nelson, MSN, RN, CNS	24008<br>Krishna Kumar Pudi, MD	1222.24 and<br>Amendment 01	29/Apr/2009<br>25/Aug/2009
Schulman Associates, IRB, 4290 Glendale–Milford Rd, Cincinnati, OH 45242	Sharon Nelson, MSN, RN, CNS	24007<br>Jose Angel Santiago, Jr., MD	1222.24 and<br>Amendment 01	29/Apr/2009<br>25/Aug/2009

Schulman Associates, IRB, 4290 Glendale–Milford Rd, Cincinnati, OH 45242	Sharon Nelson, MSN, RN, CNS	24005 Robert Walker, MD	1222.24 and Amendment 01	30/Apr/2009 25/Aug/2009
Study 1222.25				
Schulman Associates, IRB, 4290 Glendale–Milford Rd, Cincinnati, OH 45242	Sharon Nelson, MSN, RN, CNS	24014 Maxwell L. Axler, MD	1222.25 and Amendment 01	29/Apr/2009 Not needed
Schulman Associates, IRB, 4290 Glendale–Milford Rd, Cincinnati, OH 45242	Sharon Nelson, MSN, RN, CNS	24005 Ravi T. Chandran, MD	1222.25 and Amendment 01	13/May/2009 Not needed
Schulman Associates, IRB, 4290 Glendale–Milford Rd, Cincinnati, OH 45242	Sharon Nelson, MSN, RN, CNS	25002 Leonard Jay Dunn, MD	1222.25 and Amendment 01	29/Apr/2009 26/Aug/2009

Schulman Associates, IRB,<br>4290 Glendale–Milford Rd,<br>Cincinnati, OH 45242	Sharon Nelson, MSN, RN,<br>CNS	25007<br>Faisal A. Fakih, MD	1222.25 and<br>Amendment 01	29/Apr/2009<br>25/Aug/2009
Schulman Associates, IRB,<br>4290 Glendale–Milford Rd,<br>Cincinnati, OH 45242	Sharon Nelson, MSN, RN,<br>CNS	25008<br>Walter B. Forman, MD<br>Martin J. Conway, MD	1222.25 and<br>Amendment 01	15/May/2009<br>25/Aug/2009
Schulman Associates, IRB,<br>4290 Glendale–Milford Rd,<br>Cincinnati, OH 45242	Sharon Nelson, MSN, RN,<br>CNS	25013<br>Glenn M. Giessel, MD	1222.25 and<br>Amendment 01	26/Apr/2009<br>25/Aug/2009
Schulman Associates, IRB,<br>4290 Glendale–Milford Rd,<br>Cincinnati, OH 45242	Sharon Nelson, MSN, RN,<br>CNS	25003<br>John M. Hill, MD	1222.25 and<br>Amendment 01	29/Apr/2009<br>25/Aug/2009

Schulman Associates, IRB, 4290 Glendale–Milford Rd, Cincinnati, OH 45242	Sharon Nelson, MSN, RN, CNS	25004 Craig F. LaForce, MD	1222.25 and Amendment 01	04/May/2009 25/Aug/2009
Schulman Associates, IRB, 4290 Glendale–Milford Rd, Cincinnati, OH 45242	Sharon Nelson, MSN, RN, CNS	25011 Charles A. Pue, MD	1222.25 and Amendment 01	01/Jun/2009 25/Aug/2009
Schulman Associates, IRB, 4290 Glendale–Milford Rd, Cincinnati, OH 45242	Sharon Nelson, MSN, RN, CNS	25010 Stuart J. Simon, MD	1222.25 and Amendment 01	22/May/2009 25/Aug/2009
Crescent City, IRB, 2820 Canal Street, New Orleans, LA 70119	Brandon M. Wool, MD	25006 William B. Smith, MD	1222.25 and Amendment 01	08/May/2009 28/Aug/2009

Schulman Associates, IRB, 4290 Glendale–Milford Rd, Cincinnati, OH 45242	Sharon Nelson, MSN, RN, CNS	25012 Selwyn Spangenthal, MD	1222.25 and Amendment 01	05/May/2009 25/Aug/2009
Schulman Associates, IRB, 4290 Glendale–Milford Rd, Cincinnati, OH 45242	Sharon Nelson, MSN, RN, CNS	25009 Jan Hendrick Westerman, MD	1222.25 and Amendment 01	01/May/2009 25/Aug/2009

IRB = Institutional Review Board.

Table S2 Secondary efficacy end point FVC $AUC_{0–12}$ response (L) treatment comparisons after 6 weeks

		Treatment	Adjusted[a] mean (95% CI) difference from placebo at 6 weeks		
FVC $AUC_{0–12}$	n	Adjusted mean (SE)	Mean (SE)	*P* value	95% CI
Study 1222.24					
Placebo	93	-0.086 (0.036)			
Olodaterol 5 µg QD	92	0.139 (0.036)	0.226 (0.030)	< .0001	0.167, 0.284
Olodaterol 10 µg QD	91	0.142 (0.036)	0.229 (0.030)	< .0001	0.170, 0.287
Formoterol 12 µg BID	90	0.117 (0.036)	0.203 (0.030)	< .0001	0.144, 0.262
Study 1222.25					
Placebo	91	-0.032 (0.040)			
Olodaterol 5 µg QD	92	0.219 (0.040)	0.251 (0.028)	< .0001	0.195, 0.306
Olodaterol 10 µg QD	90	0.214 (0.040)	0.246 (0.028)	< .0001	0.190, 0.302
Formoterol 12 µg BID	90	0.203 (0.040)	0.235 (0.028)	< .0001	0.179, 0.291

BID = twice daily; FVC = forced vital capacity; $AUC_{0–12}$ = area under the curve from 0 to 12 h; QD = once daily; SE = standard error.

[a]Adjusted for baseline and period.

Table S3 Secondary efficacy end point FVC $AUC_{12–24}$ response (L) treatment comparisons after 6 weeks

	Treatment		Adjusted[a] mean (95% CI) difference from placebo at 6 weeks		
FVC $AUC_{12–24}$	n	Adjusted mean (SE)	Mean (SE)	*P* value	95% CI
Study 1222.24					
Placebo	93	-0.164 (0.036)			
Olodaterol 5 µg QD	92	-0.016 (0.037)	0.147 (0.030)	< .0001	0.087, 0.207
Olodaterol 10 µg QD	91	0.008 (0.037)	0.171 (0.030)	< .0001	0.112, 0.231
Formoterol 12 µg BID	90	0.088 (0.037)	0.251 (0.030)	< .0001	0.191, 0.311
Study 1222.25					
Placebo	91	-0.078 (0.041)			
Olodaterol 5 µg QD	92	0.083 (0.041)	0.161 (0.031)	< .0001	0.101, 0.222
Olodaterol 10 µg QD	90	0.079 (0.041)	0.157 (0.031)	< .0001	0.096, 0.218
Formoterol 12 µg BID	90	0.144 (0.041)	0.223 (0.031)	< .0001	0.162, 0.284

BID = twice daily; FVC = forced vital capacity; $AUC_{12–24}$ = area under the curve from 12 to 24 h; QD = once daily; SE = standard error.

[a]Adjusted for baseline and period.

Table S4 Secondary efficacy end point FVC $AUC_{0–24}$ response (L) treatment comparisons after 6 weeks

		Treatment	Adjusted[a] mean (95% CI) difference from placebo at 6 weeks		
FVC $AUC_{0–24}$	n	Adjusted mean (SE)	Mean (SE)	*P* value	95% CI
Study 1222.24					
Placebo	93	-0.125 (0.035)			
Olodaterol 5 µg QD	92	0.061 (0.035)	0.186 (0.028)	<.0001	0.132, 0.241
Olodaterol 10 µg QD	91	0.075 (0.035)	0.200 (0.028)	<.0001	0.145, 0.254
Formoterol 12 µg BID	90	0.102 (0.035)	0.227 (0.028)	<.0001	0.172, 0.282
Study 1222.25					
Placebo	91	-0.055 (0.039)			
Olodaterol 5 µg QD	92	0.151 (0.039)	0.206 (0.026)	<.0001	0.155, 0.258
Olodaterol 10 µg QD	90	0.147 (0.039)	0.202 (0.027)	<.0001	0.150, 0.255
Formoterol 12 µg BID	90	0.174 (0.039)	0.229 (0.027)	<.0001	0.176, 0.281

BID = twice daily; FVC = forced vital capacity; $AUC_{0–24}$ = area under the curve from 0 to 24 h; QD = once daily; SE = standard error.

[a]Adjusted for baseline and period.

Table S5 Secondary efficacy end point FVC peak response (L) and comparisons to placebo after 6 weeks

	Treatment		Adjusted mean (95% CI) difference from placebo at 6 weeks		
FVC peak	n	Adjusted mean (SE)	Mean (SE)	*P* value	95% CI
Study 1222.24					
Placebo	93	0.107 (0.038)			
Olodaterol 5 µg QD	92	0.368 (0.039)	0.261 (0.035)	<.0001	0.191, 0.330
Olodaterol 10 µg QD	91	0.360 (0.039)	0.252 (0.036)	<.0001	0.183, 0.322
Formoterol 12 µg BID	90	0.410 (0.039)	0.302 (0.036)	<.0001	0.232, 0.372
Study 1222.25					
Placebo	91	0.138 (0.043)			
Olodaterol 5 µg QD	92	0.436 (0.043)	0.299 (0.034)	<.0001	0.233, 0.365
Olodaterol 10 µg QD	90	0.440 (0.043)	0.302 (0.034)	<.0001	0.236, 0.369
Formoterol 12 µg BID	90	0.475 (0.043)	0.338 (0.034)	<.0001	0.271, 0.405

BID = twice daily; FVC = forced vital capacity; QD = once daily; SE = standard error.

[a]Adjusted for baseline and period.

Table S6 Secondary efficacy end point trough FVC response (L) treatment comparisons after 6 weeks

		Treatment	Adjusted[a] mean (95% CI) difference from placebo at 6 weeks		
Trough FVC	n	Adjusted mean (SE)	Mean (SE)	*P* value	95% CI
Study 1222.24					
Placebo	93	-0.117 (0.039)			
Olodaterol 5 µg QD	92	0.037 (0.040)	0.154 (0.034)	<.0001	0.087, 0.221
Olodaterol 10 µg QD	91	0.037 (0.040)	0.154 (0.034)	<.0001	0.086, 0.221
Formoterol 12 µg BID	90	0.066 (0.040)	0.183 (0.034)	<.0001	0.115, 0.250
Study 1222.25					
Placebo	91	-0.007 (0.053)			
Olodaterol 5 µg QD	92	0.125 (0.053)	0.132 (0.055)	.0163	0.024, 0.239
Olodaterol 10 µg QD	90	0.133 (0.054)	0.139 (0.055)	.0118	0.031, 0.247
Formoterol 12 µg BID	90	0.141 (0.054)	0.148 (0.055)	.0076	0.040. 0.256

BID = twice daily; FVC = forced vital capacity; QD = once daily; SE = standard error.

[a]Adjusted for baseline and period.

Table S7 AEs (pooled data)

AE	Placebo<br>(n = 190)	Olodaterol 5 µg<br>(n = 188)	Olodaterol 10 µg<br>(n = 187)	Formoterol 12 µg<br>(n = 186)
Investigator-defined drug-related AE	2 (1.1)	5 (2.7)	5 (2.7)	4 (2.2)
Serious AEs	7 (3.7)	7 (3.7)	5 (2.7)	5 (2.7)
Fatal	0	1 (0.5)	1 (0.5)	0
Immediately life-threatening	0	1 (0.5)	1 (0.5)	1 (0.5)
Incapacitating	0	0	0	0
Requiring hospitalization	6 (3.2)	5 (2.7)	4 (2.1)	4 (2.2)
Prolonging hospitalization	0	0	0	0
Other	1 (0.5)	1 (0.5)	0	0

AE = adverse event; COPD = chronic obstructive pulmonary disease.
